# Intrinsic Thalamic Network in Temporal Lobe Epilepsy With Hippocampal Sclerosis According to Surgical Outcomes

**DOI:** 10.3389/fneur.2021.721610

**Published:** 2021-08-27

**Authors:** Kyoo Ho Cho, Ho-Joon Lee, Kyoung Heo, Sung Eun Kim, Dong Ah Lee, Kang Min Park

**Affiliations:** ^1^Department of Neurology, Yonsei University College of Medicine, Seoul, South Korea; ^2^Department of Neurology, Seoul Hospital, Ewha Womans University College of Medicine, Seoul, South Korea; ^3^Department of Radiology, Haeundae Paik Hospital, Inje University College of Medicine, Busan, South Korea; ^4^Department of Neurology, Haeundae Paik Hospital, Inje University College of Medicine, Busan, South Korea

**Keywords:** thalamus, network, epilepsy, seizure, surgery

## Abstract

**Background:** The aim of this study was to identify the differences of intrinsic amygdala, hippocampal, or thalamic networks according to surgical outcomes in temporal lobe epilepsy (TLE) patients with hippocampal sclerosis (HS).

**Methods:** We enrolled 69 pathologically confirmed TLE patients with HS. All patients had pre-operative three-dimensional T1-weighted MRI using a 3.0 T scanner. We obtained the structural volumes of the amygdala nuclei, hippocampal subfields, and thalamic nuclei. Then, we investigated the intrinsic networks based on volumes of these structures using structural covariance and graph theoretical analysis.

**Results:** Of the 69 TLE patients with HS, 21 patients (42.1%) had poor surgical outcomes, whereas 40 patients (57.9%) had good surgical outcomes. The volumes in the amygdala nuclei, hippocampal subfields, and thalamic nuclei were not different according to surgical outcome. In addition, the intrinsic amygdala and hippocampal networks were not different between the patients with poor and good surgical outcomes. However, there was a significant difference in the intrinsic thalamic network in the ipsilateral hemisphere between them. The eccentricity and small-worldness index were significantly increased, whereas the characteristic path length was decreased in the patients with poor surgical outcomes compared to those with good surgical outcomes.

**Conclusion:** We successfully demonstrated significant differences in the intrinsic thalamic network in the ipsilateral hemisphere between TLE patients with HS with poor and good surgical outcomes. This result suggests that the pre-operative intrinsic thalamic network can be related with surgical outcomes in TLE patients with HS.

## Introduction

Temporal lobe epilepsy (TLE) with hippocampal sclerosis (HS) is the most common cause of drug-resistant focal epilepsy in adults (1). In the cases of patients with drug-resistant focal epilepsy, especially TLE patients with HS, epileptic surgery can be helpful for controlling epileptic seizures (2). Although the several epileptic surgical techniques, anterior temporal lobectomy is the most common and standard surgical method in the treatment of TLE patients with HS (3). After anterior temporal lobectomy in TLE patients with HS, approximately one-third of patients cannot achieve seizure-free status (2).

There have been many studies on the predictive factors for poor surgical outcome after anterior temporal lobectomy in TLE patients with HS, which define patients who will benefit the most from surgery. Clinical factors, such as old age at surgery, focal to bilateral tonic-clonic seizures, or bitemporal interictal epileptiform discharges, have been associated with seizure recurrence after surgery (4–6). In addition, emerging evidence has suggested that post-processing pre-operative brain magnetic resonance imaging (MRI) shed light to predict surgical outcomes for TLE patients with HS. A previous study demonstrated that gray matter abnormalities in the entorhinal cortex and posterior parahippocampal gyrus beyond HS are a poor prognostic factor for poor seizure outcome in TLE patients with HS (7). Another study revealed that volume loss in cornu amonis (CA) 1 and 4 is related to good surgical outcomes (8). In addition, alteration of thalamic nuclei was reported to participate in seizure modulation and propagation (9).

Focal seizures are conceptualized as originating within networks limited to one hemisphere, and the concept of focal epilepsy, including TLE with HS, is now considered a network disease that has recurrent focal seizures (10, 11). One of the hypotheses underlying surgical failure in TLE with HS is that an occult epileptogenic network encompassing extra-hippocampal structures is already established and spared after surgery (12). The amygdala, thalamus, and hippocampus could be epileptogenic hubs in TLE patients with HS. These nodes can be a potential target for the treatment of TLE with HS. Recently, we can obtain the volumes of the structures and therefore calculate the intrinsic amygdala, hippocampal, and thalamic networks based on these volumes using structural covariance analysis (13, 14). However, there have been no studies investigating whether the intrinsic amygdala, hippocampal, or thalamic networks can be possible biomarkers for predicting surgical outcomes in TLE patients with HS.

We hypothesized that identification of differences in intrinsic amygdala, hippocampal, or thalamic networks using pre-surgical MRI could help uncover novel biomarkers for predicting surgical outcomes in TLE patients with HS. Thus, we calculated the intrinsic amygdala, hippocampal, and thalamic networks using pre-operative MRI in TLE patients with HS and investigated their differences between patients with poor and good surgical outcomes.

## Methods

### Participants

This study was approved by our Institutional Review Board. This was a retrospective study conducted at a tertiary hospital. We enrolled TLE patients with HS according to the following criteria: (1) typical seizure semiology compatible with mesial TLE, (2) ictal EEG originating from the anterior temporal lobe, (3) typical MRI findings of HS, including increased signal intensity on FLAIR with decreased volume, (4) standard anterior temporal lobectomy from January 2010 to December 2019, (5) pathologically confirmed HS, and (6) at least 12 months of follow-up after surgery. We obtained the clinical characteristics, including age, sex, age of seizure onset, duration of epilepsy (time from seizure onset to surgery), history of febrile seizure, presence of focal to bilateral tonic-clonic seizures, and seizure frequency (number of focal onset seizures with or without bilateral tonic-clonic seizures during the year before surgery). We excluded patients with any lesions other than HS on brain MRI or bilateral HS. We classified the patients into two groups: patients with poor surgical outcomes (ILAE III-VI) and those with good surgical outcomes (ILAE I-II) (15).

### Acquisition of Brain MRI

All patients had pre-operative 3D T1-weighted MRI using a 3.0T scanner (Achieva Tx, Philips Healthcare, Best, the Netherlands) equipped with a 32 channel head coil. The 3D T1 turbo field echo acquisition was performed in the coronal plane, with the following parameters: echo time (TE) 4.6 ms, repetition time (TR) 9.6 ms, flip angle 8°, and 1mm^3^ isotropic voxel size.

### Obtaining the Volumes in the Amygdala Nuclei, Hippocampal Subfields, and Thalamic Nuclei

We obtained volumetric measures of each subjects' MRI using the FreeSurfer program (version 7), which produced an automatic segmentation of the amygdala nuclei, hippocampal subfields, and thalamic nuclei. This tool uses a probabilistic atlas built with ultra-high resolution MRI data. We followed the main FreeSurfer stream (“recon-all”) and used the commands “segmentHA_T1” and “segment Thalamic Nuclei” to obtain the volumes of the amygdala nuclei, hippocampal subfields, and thalamic nuclei (16, 17). This tool produced a parcellation of the amygdala into nine nuclei, the hippocampus into 21 subfields, and the thalamus into 25 nuclei (Supplementary Material 1). All segmentations were visually inspected for accuracy prior to inclusion in the group analysis to correct potential error during automated procedure. We corrected the absolute structural volumes with an individual total intracranial volume. We defined the ipsilateral hemisphere as the side of the HS, whereas the contralateral hemisphere as the contralateral side of the HS.

### Intrinsic Amygdala, Hippocampal, and Thalamic Network

**Figure 1:**
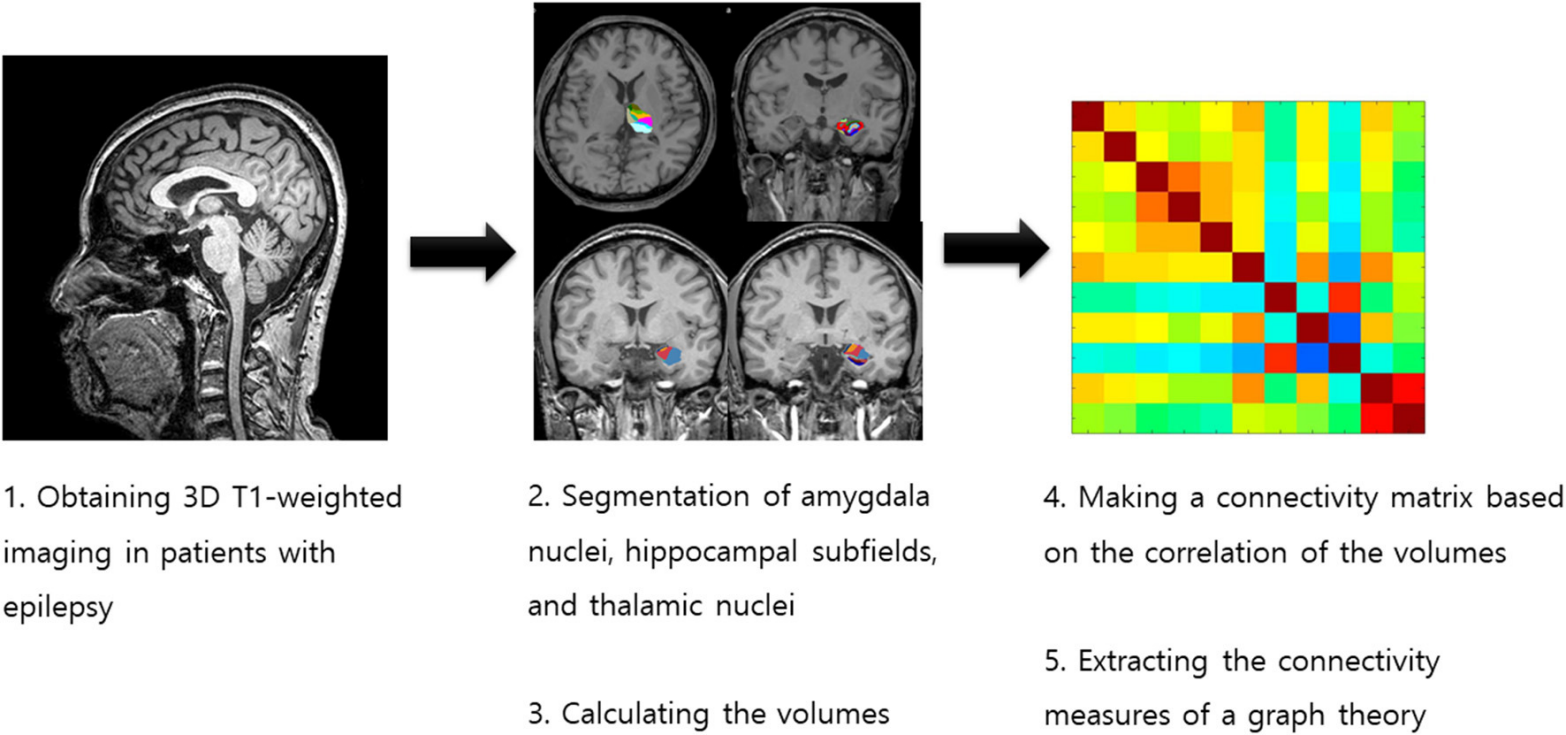
Process for obtaining the intrinsic amygdala, hippocampal, and thalamic networks in temporal lobe epilepsy patients with hippocampal sclerosis.

We investigated the intrinsic amygdala, hippocampal, and thalamic networks based on volume using structural covariance analysis and graph theory (Figure 1). We calculated the network measures of graph theory using the BRAPH toolbox (18). We defined the nodes as the volumes of the amygdala nuclei, hippocampal subfields, and thalamic nuclei and the edges as the partial correlation coefficient while controlling the effects of age and sex. We made a weighted connectivity matrix and extracted the network measures from the matrix, such as average strength, radius, diameter, eccentricity, characteristic path length, global efficiency, local efficiency, mean clustering coefficient, transitivity, modularity, assortative coefficient, and the small-worldness index (18, 19). We investigated the differences in these network measures between patients with poor and good surgical outcomes.

### Statistical Analysis

The statistical analysis of the clinical characteristics between the groups was performed using the chi-squared test for categorical variables and the Student's *t*-test or Mann-Whitney test depending on normal distribution or not for numerical variables. In the comparison of the network measures, we used non-parametric permutation tests with 1,000 permutations because we could obtain the measures as a group level. A *p*-value < 0.05 indicated statistical significance in all analyses. We conducted a multiple correction with Bonferroni correction when we investigated the differences in the volume and network (amygdala nuclei volume, *p* < 0.002 (0.05/18, total 18 numbers of amygdala nuclei); hippocampal subfields volume, *p* < 0.001 (0.05/42, total 42 numbers of hippocampal subfields); thalamic nuclei volume, *p* < 0.001 (0.05/50, total 50 numbers of thalamic nuclei), network measures, *p* < 0.004 (0.05/12, total 12 numbers of network measures). All statistical tests were performed using MedCalc® (MedCalc Software version 19.4.0, Ostend, Belgium; https://www.medcalc.org; 2020).

## Results

### Clinical Characteristics of the Patients With Epilepsy

We enrolled 69 TLE patients with HS. The mean age at the time of surgery was 36.8 ± 10.5 years, and the median age of seizure onset was 14 years. The median seizure frequency was 24/year, and the median follow-up period after surgery was 96 months. Of the 69 TLE patients with HS, 32 patients (46.3%) had right HS, whereas 37 patients (53.6%) had left HS. Twenty-six patients (37.6%) were men, whereas 43 patients (62.3%) were women. Twenty-nine patients (42.1%) had poor surgical outcomes, whereas 40 patients (57.9%) had good surgical outcomes (30 patients with ILAE I, 10 patients with ILAE II, 19 patients with ILAE III, three patients with ILAE IV, five patients with ILAE V, and two patients with ILAE VI). Table 1 shows the differences in the clinical characteristics of the TLE patients with HS according to surgical outcome. The clinical characteristics, including age at the time of surgery, sex, age of seizure onset, history of febrile seizures, presence of focal to bilateral tonic-clonic seizures, seizure frequency, side of HS, and follow-up period after surgery, were not different between the patients with poor and good surgical outcomes.

**Table 1:** Clinical characteristics of temporal lobe epilepsy patients with hippocampal sclerosis according to surgical outcome.

	**Patients with poor surgical outcome, *N* = 29**	**Patients with good surgical outcome, *N* = 40**	***p*-value**
Mean age at the time of surgery, years	38.7 ± 9.0	35.4 ± 11.3	0.198
Male, *N* (%)	12 (41.3)	14 (35.0)	0.592
Median age of seizure onset, years (interquartile range)	10 (4–18)	16 (9–26)	0.063
Duration of epilepsy, months (interquartile range)	295 (142–422)	234 (148–369)	0.458
History of febrile seizure, *N* (%)	7 (24.1)	13 (32.5)	0.453
Presence of focal to bilateral tonic-clonic seizures, *N* (%)	21 (72.4)	27 (67.5)	0.663
Median seizure frequency, *N*/year (interquartile range)	24 (14–72)	24 (15–39)	0.502
Right HS, *N* (%)	10 (34.4)	22 (55.0)	0.094
Median follow-up periods after surgery, months (interquartile range)	108 (72–144)	90 (60–126)	0.227

### Volumes in the Amygdala Nuclei, Hippocampal Subfields, and Thalamic Nuclei

Supplementary Material 1 shows the volumetric measures of the amygdala nuclei, hippocampal subfields, and thalamic nuclei in TLE patients with HS. There were noted the differences between the patients with poor and good surgical outcomes before multiple corrections, such as anteroventral, central medial, paratenial, pulvinar lateral, ventral anterior, ventral anterior magnocellular, ventral lateral posterior, and ventral posterolateral thalamic nuclei; hippocampal tail in the ipsilateral hemisphere; and reuniens and pulvinar inferior thalamic nuclei in the contralateral hemisphere before multiple corrections (Table 2). However, none of them showed significant differences after multiple corrections.

**Table 2:** Volumes of the amygdala nuclei, hippocampal subfields, and thalamic nuclei in temporal lobe epilepsy patients with hippocampal sclerosis according to surgical outcome.

	**Poor surgical outcome**		**Good surgical outcome**			
	**Mean (%)**	**SD (%)**	**Mean (%)**	**SD (%)**	**Difference (%)**	***p*-value**
**Ipsilateral hemisphere**						
Anteroventral thalamic nucleus	0.0059	0.0019	0.0070	0.0017	−0.0011	0.012
Central medial thalamic nucleus	0.0033	0.0007	0.0038	0.0008	−0.0005	0.008
Hippocampal tail	0.0289	0.0086	0.0335	0.0099	−0.0047	0.046
Paratenial thalamic nucleus	0.0004	0.0001	0.0004	0.0001	0.0000	0.022
Pulvinar lateral thalamic nucleus	0.0127	0.0026	0.0144	0.0023	−0.0017	0.006
Ventral anterior thalamic nucleus	0.0223	0.0046	0.0247	0.0039	−0.0024	0.024
Ventral anterior magnocellular thalamic nucleus	0.0018	0.0003	0.0020	0.0003	−0.0002	0.016
Ventral lateral anterior thalamic nucleus	0.0362	0.0075	0.0399	0.0059	−0.0037	0.025
Ventral lateral posterior thalamic nucleus	0.0471	0.0098	0.0519	0.0079	−0.0047	0.029
Ventral posterolateral thalamic nucleus	0.0496	0.0088	0.0544	0.0101	−0.0047	0.048
**Contralateral hemisphere**						
Reuniens thalamic nucleus	0.0006	0.0002	0.0006	0.0001	−0.0001	0.050
Pulvinar inferior thalamic nucleus	0.0135	0.0023	0.0150	0.0028	−0.0016	0.015

### Intrinsic Amygdala, Hippocampal, and Thalamic Networks

Supplementary Material 2 shows the differences in the intrinsic amygdala, hippocampal, and thalamic networks in TLE patients with HS according to surgical outcome. The intrinsic amygdala and hippocampal networks in the ipsilateral and contralateral hemispheres and the intrinsic thalamic network in the contralateral hemisphere were not different between the patients with poor and good surgical outcomes. However, there was a significant difference in the intrinsic thalamic network in the ipsilateral hemisphere between them. The eccentricity and small-worldness index were significantly increased (4.792 vs. 3.757, *p* = 0.001; 0.976 vs. 0.952, *p* = 0.001, respectively), whereas the characteristic path length was decreased in the patients with poor surgical outcomes compared to those with good surgical outcomes (1.751 vs. 1.999, *p* = 0.001).

## Discussion

In this study, we demonstrated significant differences in the intrinsic thalamic network in the ipsilateral hemisphere between TLE patients with HS with poor and good surgical outcomes. However, the other intrinsic networks, including the intrinsic amygdala and hippocampus in the ipsilateral and contralateral hemispheres and the intrinsic thalamic network in the contralateral hemisphere, were not different according to surgical outcomes. In addition, we found that the volumes of the amygdala nuclei, hippocampal subfields, and thalamic nuclei in the ipsilateral and contralateral hemispheres were not different between the patients with poor and good surgical outcomes.

In the present study, we demonstrated that brain connectivity could predict surgical outcomes in TLE with HS. These results suggest that the use of connectivity could have a significant impact on pre-surgical evaluations in clinical practice. There were several previous studies in line with our results. Morgan et al. computed a model of pre-surgical MRI-based functional and structural connectivity using functional MRI and diffusion tensor imaging that successfully separated TLE patients with poor and good surgical outcomes (20). Another study on the basis of pre-surgical functional MRI connectivity showed a specific increase in nodal hubness involving both the ipsilateral and contralateral thalamus in patients with poor surgical outcomes compared to those with good surgical outcomes (21). They also used a machine learning approach based on functional connectivity features and demonstrated 74% accuracy in predicting seizure outcome, which exceeded the predictive value of models with solely clinical characteristics (21). Furthermore, brain connectivity based on other modalities could be useful for predicting surgical outcomes. A study with magnetoencephalography revealed that patients with increased regional connectivity within the resection site were more likely to achieve good surgical outcomes than those with decreased regional connectivity (22). The seizure outcome of vagal nerve stimulation was also influenced by metabolic connectivity obtained from pre-operative fluoro-D-glucose positron emission tomography imaging (23). A significant difference in metabolic connectivity was noted between poor and good surgical outcomes (23). All of these studies suggested that brain connectivity analysis probably help predict post-operative seizure outcomes and aid surgical planning.

With a graph theoretical analysis, we found that there were significant differences in the ipsilateral intrinsic thalamic network according to surgical outcomes. The patients with poor surgical outcomes had increased eccentricity and small-worldness index with a decrease in characteristic path length in the ipsilateral intrinsic thalamic network. Graph theory is a branch of mathematics that provides a complete map of all relations among nodes (collection of elements) and edges (pairwise links) (24). Path length is the average distance from a node to all other nodes, and the characteristic path length is the average of the path lengths of all nodes (18, 25). A small-world graph has a similar characteristic path length as a random graph with the same degree distribution but is significantly more clustered. The eccentricity is the maximal distance between a certain node and any other node (18, 25). Therefore, an increased small-worldness index with decreased characteristic path length means high global and local efficiency and the ability of the entire network to transfer information in parallel, which reveals high integration and segregation of the network (26). It is characterized by a high speed of information diffusion and ease of resource mobilization, suggesting an increased connectivity of the network (26).

TLE with HS has alterations associated with a redistribution of hubness, which resided in structures of epileptic network (27, 28). The thalamus is well recognized for its pivotal role in the generation and spreading of seizures in TLE with HS (29, 30). In addition, the thalamus is a crucial hub for cortical synchrony and modulation of brain rhythm. Thus, it is a hub in the potential epileptogenic network in TLE with HS (21). A previous study demonstrated that ipsilateral intrathalamic connectivity and thalamic volume were reduced and that connectivity was impaired in thalamic arousal networks (31). Keller et al. also found that TLE patients with HS had significant atrophy of the bilateral dorsomedial and pulvinar thalamic regions and significant alterations in thalamo-temporal probabilistic paths relative to those with good surgical outcomes (32). Another study based on EEG found that patients with good surgical outcomes had low values of thalamo-cortical couplings at seizure onset compared to those with poor surgical outcomes (33). Furthermore, the patients with poor surgical outcomes displayed an increase in degree and eigenvector centralities involving both thalami using functional MRI (21). All of these results of previous studies and our present findings indicate that the thalamus has a pivotal role as a hub of the epileptogenic network in TLE with HS and is crucial to seizure recurrence after surgery. This finding is in agreement with a SANTE trial (Stimulation of the Anterior Nucleus of Thalamus for Epilepsy), revealing that deep brain stimulation of the anterior thalamus is useful for patients with medically refractory focal seizures, especially TLE (34). However, we found that the intrinsic amygdala and hippocampus networks were not different according to surgical outcomes. We could not exactly know the reason for these findings. However, we can assume that TLE occurs because of alterations of temporal lobe network, including intrinsic amygdala and hippocampus networks, and network abnormality beyond temporal structure, such as intrinsic thalamic network, is more related with the success of the anterior temporal lobectomy in patients with TLE (35, 36).

There were several limitations for this study. First, this was a retrospective study performed at a tertiary hospital. To demonstrate reproducibility, further prospective studies in multiple centers with large sample sizes are needed. Second, surgical outcome varies over time. Long term outcome remains to be explored. Third, in terms of method, with recent advances in MRI scanners and analytic software programs, we obtained the structural volumes of the amygdala nuclei, hippocampal subfields, and thalamic nuclei by brain MRI. However, further validations of the intrinsic amygdala, hippocampal, and thalamic networks based on the volumes of amygdala nuclei, hippocampal subfields, and thalamic nuclei using covariance might be needed.

We successfully demonstrated significant differences in the intrinsic thalamic network in the ipsilateral hemisphere between TLE patients with HS with poor and good surgical outcomes. This result suggests that the pre-operative intrinsic thalamic network can be related with surgical outcomes in TLE patients with HS.

## Data Availability Statement

The original contributions presented in the study are included in the article/Supplementary Material, further inquiries can be directed to the corresponding author/s.

## Ethics Statement

The studies involving human participants were reviewed and approved by Yonsei University College of Medicine, Seoul, South Korea. Written informed consent for participation was not required for this study in accordance with the national legislation and the institutional requirements.

## Author Contributions

KC and H-JL: participated in data collection, data interpretation, and manuscript writing. KH, SK, and DL: participated in data collection and data interpretation. KP: supervised analysis, participated in study design, data interpretation, and manuscript writing. All authors provided critical feedback, read, and approved the final manuscript.

## Conflict of Interest

The authors declare that the research was conducted in the absence of any commercial or financial relationships that could be construed as a potential conflict of interest.

## Publisher's Note

All claims expressed in this article are solely those of the authors and do not necessarily represent those of their affiliated organizations, or those of the publisher, the editors and the reviewers. Any product that may be evaluated in this article, or claim that may be made by its manufacturer, is not guaranteed or endorsed by the publisher.
