# Supplementary material for: Intrinsic Thalamic Network in Temporal Lobe Epilepsy With Hippocampal Sclerosis According to Surgical Outcomes
Source: Front Neurol. 2021 Aug 27;12:721610. doi: 10.3389/fneur.2021.721610 (PMC8429827; doi:10.3389/fneur.2021.721610)
Supplement: Supplementary file 1 [file Table_1.DOCX]

**Supplementary 1.** Volumes of the amygdala nuclei, hippocampal subfields, and thalamic nuclei in temporal lobe epilepsy patients with hippocampal sclerosis according to surgical outcome

| Regions | Patients with poor surgical outcome | | Patients with good surgical outcome | |  |  | Patients with poor surgical outcome | | Patients with good surgical outcome | |  |
| --- | --- | --- | --- | --- | --- | --- | --- | --- | --- | --- | --- |
|  | Mean (%) | SD (%) | Mean (%) | SD (%) | *p*-value |  | Mean (%) | SD (%) | Mean (%) | SD (%) | *p*-value |
| **Ipsilateral hemisphere** |  |  |  |  |  | **Contralateral hemisphere** |  |  |  |  |  |
| **Whole amygdala** | 0.043 | 0.048 | 0.073 | 0.053 | 0.020 | **Whole amygdala** | 0.084 | 0.055 | 0.056 | 0.055 | 0.039 |
| Anterior amygdala area | 0.003 | 0.001 | 0.004 | 0.001 | 0.063 | Anterior amygdala area | 0.004 | 0.001 | 0.004 | 0.001 | 0.983 |
| Cortico-amygdaloid transition area | 0.011 | 0.003 | 0.012 | 0.002 | 0.181 | Cortico-amygdaloid transition area | 0.012 | 0.002 | 0.012 | 0.002 | 0.744 |
| Lateral nucleus | 0.041 | 0.008 | 0.043 | 0.008 | 0.192 | Lateral nucleus | 0.046 | 0.006 | 0.046 | 0.006 | 0.546 |
| Basal nucleus | 0.027 | 0.006 | 0.029 | 0.005 | 0.186 | Basal nucleus | 0.031 | 0.004 | 0.031 | 0.004 | 0.726 |
| Paralaminar nucleus | 0.003 | 0.001 | 0.003 | 0.001 | 0.224 | Paralaminar nucleus | 0.003 | 0.000 | 0.003 | 0.000 | 0.838 |
| Accessory basal nucleus | 0.016 | 0.004 | 0.017 | 0.003 | 0.319 | Accessory basal nucleus | 0.019 | 0.003 | 0.019 | 0.003 | 0.807 |
| Medial nucleus | 0.001 | 0.000 | 0.001 | 0.000 | 0.207 | Medial nucleus | 0.001 | 0.000 | 0.001 | 0.000 | 0.656 |
| Central nucleus | 0.003 | 0.001 | 0.003 | 0.001 | 0.116 | Central nucleus | 0.003 | 0.001 | 0.003 | 0.001 | 0.958 |
| Cortical nucleus | 0.002 | 0.000 | 0.002 | 0.000 | 0.474 | Cortical nucleus | 0.002 | 0.000 | 0.002 | 0.000 | 0.782 |
| **Whole hippocampus** | 0.183 | 0.056 | 0.200 | 0.053 | 0.188 | **Whole hippocampus** | 0.233 | 0.037 | 0.240 | 0.037 | 0.455 |
| CA1 body | 0.006 | 0.002 | 0.007 | 0.002 | 0.171 | CA1 body | 0.008 | 0.002 | 0.008 | 0.002 | 0.494 |
| CA1 head | 0.028 | 0.009 | 0.030 | 0.008 | 0.272 | CA1 head | 0.036 | 0.006 | 0.036 | 0.006 | 0.990 |
| CA3 body | 0.005 | 0.001 | 0.005 | 0.002 | 0.146 | CA3 body | 0.006 | 0.001 | 0.006 | 0.001 | 0.885 |
| CA3 head | 0.006 | 0.002 | 0.007 | 0.002 | 0.375 | CA3 head | 0.008 | 0.002 | 0.009 | 0.002 | 0.649 |
| CA4 body | 0.006 | 0.002 | 0.007 | 0.002 | 0.399 | CA4 body | 0.008 | 0.001 | 0.008 | 0.002 | 0.372 |
| CA4 head | 0.006 | 0.003 | 0.007 | 0.002 | 0.398 | CA4 head | 0.009 | 0.002 | 0.009 | 0.002 | 0.627 |
| Fimbria | 0.005 | 0.001 | 0.005 | 0.002 | 0.104 | Fimbria | 0.005 | 0.001 | 0.006 | 0.001 | 0.295 |
| Granule cell layer of dentate gyrus body | 0.007 | 0.002 | 0.008 | 0.002 | 0.321 | Granule cell layer of dentate gyrus body | 0.009 | 0.002 | 0.009 | 0.002 | 0.365 |
| Granule cell layer of dentate gyrus head | 0.008 | 0.003 | 0.009 | 0.003 | 0.314 | Granule cell layer of dentate gyrus head | 0.011 | 0.002 | 0.011 | 0.002 | 0.628 |
| Hippocampal amygdala transition area | 0.004 | 0.001 | 0.004 | 0.001 | 0.460 | Hippocampal amygdala transition area | 0.004 | 0.001 | 0.004 | 0.001 | 0.706 |
| Hippocampal fissure | 0.009 | 0.002 | 0.010 | 0.002 | 0.585 | Hippocampal fissure | 0.010 | 0.002 | 0.010 | 0.002 | 0.981 |
| Hippocampal tail | 0.029 | 0.009 | 0.034 | 0.010 | 0.046 | Hippocampal tail | 0.037 | 0.006 | 0.040 | 0.008 | 0.120 |
| Molecular layer hippocampal body | 0.012 | 0.004 | 0.013 | 0.004 | 0.150 | Molecular layer hippocampal body | 0.015 | 0.002 | 0.015 | 0.003 | 0.524 |
| Molecular layer hippocampal head | 0.018 | 0.006 | 0.019 | 0.005 | 0.294 | Molecular layer hippocampal head | 0.023 | 0.004 | 0.023 | 0.004 | 0.817 |
| Parasubiculum | 0.003 | 0.001 | 0.003 | 0.001 | 0.483 | Parasubiculum | 0.004 | 0.001 | 0.004 | 0.001 | 0.583 |
| Presubiculum body | 0.009 | 0.003 | 0.010 | 0.003 | 0.294 | Presubiculum body | 0.011 | 0.003 | 0.011 | 0.003 | 0.912 |
| Presubiculum head | 0.008 | 0.002 | 0.008 | 0.002 | 0.357 | Presubiculum head | 0.009 | 0.001 | 0.009 | 0.001 | 0.767 |
| Subiculum body | 0.013 | 0.004 | 0.014 | 0.004 | 0.380 | Subiculum body | 0.016 | 0.003 | 0.017 | 0.004 | 0.271 |
| Subiculum head | 0.011 | 0.003 | 0.012 | 0.003 | 0.494 | Subiculum head | 0.013 | 0.002 | 0.014 | 0.002 | 0.891 |
| Hippocampal body | 0.062 | 0.018 | 0.068 | 0.019 | 0.194 | Hippocampal body | 0.078 | 0.013 | 0.081 | 0.015 | 0.425 |
| Hippocampal head | 0.092 | 0.030 | 0.099 | 0.026 | 0.313 | Hippocampal head | 0.118 | 0.020 | 0.119 | 0.018 | 0.794 |
| **Whole thalamus** | 0.372 | 0.066 | 0.405 | 0.058 | 0.031 | **Whole thalamus** | 0.404 | 0.045 | 0.419 | 0.054 | 0.235 |
| Anteroventral nucleus | 0.006 | 0.002 | 0.007 | 0.002 | 0.012 | Anteroventral nucleus | 0.007 | 0.001 | 0.007 | 0.001 | 0.565 |
| Central medial nucleus | 0.003 | 0.001 | 0.004 | 0.001 | 0.008 | Central medial nucleus | 0.004 | 0.001 | 0.004 | 0.001 | 0.093 |
| Central lateral nucleus | 0.001 | 0.000 | 0.002 | 0.001 | 0.059 | Central lateral nucleus | 0.002 | 0.000 | 0.002 | 0.000 | 0.534 |
| Centromedian nucleus | 0.014 | 0.002 | 0.016 | 0.003 | 0.069 | Centromedian nucleus | 0.016 | 0.002 | 0.016 | 0.003 | 0.238 |
| Limitans nucleus | 0.001 | 0.000 | 0.001 | 0.000 | 0.627 | Limitans nucleus | 0.001 | 0.000 | 0.001 | 0.000 | 0.265 |
| Laterodorsal nucleus | 0.001 | 0.000 | 0.001 | 0.001 | 0.066 | Laterodorsal nucleus | 0.001 | 0.000 | 0.001 | 0.000 | 0.151 |
| Lateral geniculate nucleus | 0.012 | 0.002 | 0.013 | 0.002 | 0.144 | Lateral geniculate nucleus | 0.013 | 0.002 | 0.013 | 0.002 | 0.132 |
| Lateral posterior nucleus | 0.006 | 0.002 | 0.007 | 0.001 | 0.184 | Lateral posterior nucleus | 0.007 | 0.001 | 0.007 | 0.001 | 0.107 |
| Mediodorsal lateral parvocellular nucleus | 0.015 | 0.003 | 0.016 | 0.003 | 0.188 | Mediodorsal lateral parvocellular nucleus | 0.017 | 0.002 | 0.017 | 0.002 | 0.725 |
| Mediodorsal medial magnocellular nucleus | 0.043 | 0.009 | 0.046 | 0.008 | 0.173 | Mediodorsal medial magnocellular nucleus | 0.046 | 0.008 | 0.048 | 0.007 | 0.298 |
| Medial geniculate nucleus | 0.007 | 0.001 | 0.007 | 0.001 | 0.145 | Medial geniculate nucleus | 0.008 | 0.001 | 0.008 | 0.001 | 0.746 |
| Reuniens nucleus | 0.001 | 0.000 | 0.001 | 0.000 | 0.335 | Reuniens nucleus | 0.001 | 0.000 | 0.001 | 0.000 | 0.050 |
| Paracentral nucleus | 0.000 | 0.000 | 0.000 | 0.000 | 0.065 | Paracentral nucleus | 0.000 | 0.000 | 0.000 | 0.000 | 0.180 |
| Parafascicular nucleus | 0.004 | 0.001 | 0.004 | 0.001 | 0.338 | Parafascicular nucleus | 0.004 | 0.001 | 0.004 | 0.001 | 0.489 |
| Paratenial nucleus | 0.000 | 0.000 | 0.000 | 0.000 | 0.022 | Paratenial nucleus | 0.000 | 0.000 | 0.000 | 0.000 | 0.489 |
| Pulvinar anterior nucleus | 0.013 | 0.002 | 0.014 | 0.002 | 0.162 | Pulvinar anterior nucleus | 0.014 | 0.002 | 0.014 | 0.002 | 0.474 |
| Pulvinar inferior nucleus | 0.013 | 0.003 | 0.014 | 0.002 | 0.006 | Pulvinar inferior nucleus | 0.013 | 0.002 | 0.015 | 0.003 | 0.015 |
| Pulvinar lateral nucleus | 0.011 | 0.002 | 0.012 | 0.002 | 0.280 | Pulvinar lateral nucleus | 0.012 | 0.003 | 0.012 | 0.002 | 0.960 |
| Pulvinar medial nucleus | 0.062 | 0.011 | 0.067 | 0.011 | 0.059 | Pulvinar medial nucleus | 0.066 | 0.010 | 0.071 | 0.012 | 0.063 |
| Ventral anterior | 0.022 | 0.005 | 0.025 | 0.004 | 0.024 | Ventral anterior | 0.025 | 0.003 | 0.025 | 0.004 | 0.423 |
| Ventral anterior magnocellular nucleus | 0.002 | 0.000 | 0.002 | 0.000 | 0.016 | Ventral anterior magnocellular nucleus | 0.002 | 0.000 | 0.002 | 0.000 | 0.097 |
| Ventral lateral anterior nucleus | 0.036 | 0.008 | 0.040 | 0.006 | 0.025 | Ventral lateral anterior nucleus | 0.040 | 0.005 | 0.041 | 0.006 | 0.728 |
| Ventral lateral posterior nucleus | 0.047 | 0.010 | 0.052 | 0.008 | 0.029 | Ventral lateral posterior nucleus | 0.052 | 0.006 | 0.053 | 0.008 | 0.671 |
| Ventromedial nucleus | 0.001 | 0.000 | 0.001 | 0.000 | 0.052 | Ventromedial nucleus | 0.001 | 0.000 | 0.001 | 0.000 | 0.503 |
| Ventral posterolateral nucleus | 0.050 | 0.009 | 0.054 | 0.010 | 0.048 | Ventral posterolateral nucleus | 0.055 | 0.007 | 0.056 | 0.008 | 0.603 |
