# Supplementary material for: Intrinsic Thalamic Network in Temporal Lobe Epilepsy With Hippocampal Sclerosis According to Surgical Outcomes
Source: Front Neurol. 2021 Aug 27;12:721610. doi: 10.3389/fneur.2021.721610 (PMC8429827; doi:10.3389/fneur.2021.721610)
Supplement: Supplementary file 2 [file Table_2.DOCX]

**Supplementary 2.** Intrinsic amygdala, hippocampal, and thalamic networks in temporal lobe epilepsy patients with hippocampal sclerosis according to surgical outcome

|  | **Ipsilateral intrinsic amygdala network** | | | | | | **Contralateral intrinsic amygdala network** | | | | | |
| --- | --- | --- | --- | --- | --- | --- | --- | --- | --- | --- | --- | --- |
|  | Poor surgical outcome | Good surgical outcome | Difference | CI lower | CI upper | *p*-value | Poor surgical outcome | Good surgical outcome | Difference | CI lower | CI upper | *p*-value |
| Average strength | 6.526 | 6.130 | -0.396 | -1.062 | 1.099 | 0.619 | 5.714 | 5.554 | -0.160 | -0.997 | 0.995 | 0.773 |
| Radius | 1.716 | 1.473 | -0.243 | -0.556 | 0.423 | 0.451 | 1.776 | 1.805 | 0.029 | -0.540 | 0.559 | 0.968 |
| Diameter | 2.884 | 2.166 | -0.718 | -1.591 | 1.420 | 0.552 | 2.244 | 3.326 | 1.082 | -1.483 | 1.585 | 0.300 |
| Eccentricity | 2.240 | 1.856 | -0.384 | -1.157 | 1.100 | 0.577 | 1.987 | 2.467 | 0.480 | -1.011 | 0.949 | 0.422 |
| Characteristic path length | 1.333 | 1.363 | 0.030 | -0.372 | 0.323 | 0.895 | 1.462 | 1.560 | 0.097 | -0.393 | 0.330 | 0.625 |
| Global efficiency | 0.816 | 0.766 | -0.050 | -0.120 | 0.132 | 0.598 | 0.714 | 0.695 | -0.020 | -0.120 | 0.114 | 0.820 |
| Local efficiency | 1.899 | 1.681 | -0.218 | -0.578 | 0.586 | 0.603 | 1.457 | 1.387 | -0.070 | -0.502 | 0.443 | 0.881 |
| Clustering coefficient | 0.804 | 0.757 | -0.047 | -0.137 | 0.166 | 0.601 | 0.706 | 0.681 | -0.025 | -0.133 | 0.142 | 0.768 |
| Transitivity | 1.206 | 1.136 | -0.070 | -0.206 | 0.231 | 0.579 | 1.060 | 1.022 | -0.038 | -0.195 | 0.199 | 0.814 |
| Modularity | 0.000 | 0.000 | 0.000 | -0.019 | 0.005 | 0.666 | 0.000 | 0.000 | 0.000 | -0.020 | 0.012 | 0.775 |
| Assortative coefficient | -0.125 | -0.125 | 0.000 | 0.000 | 0.000 | 0.623 | -0.125 | -0.125 | 0.000 | 0.000 | 0.000 | 0.579 |
| Small-worldness index | 0.990 | 1.000 | 0.010 | -0.034 | 0.046 | 0.598 | 1.001 | 0.978 | -0.023 | -0.035 | 0.035 | 0.318 |
|  | **Ipsilateral intrinsic hippocampal network** | | | | | | **Contralateral intrinsic hippocampal network** | | | | | |
|  | Poor surgical outcome | Good surgical outcome | Difference | CI lower | CI upper | *p*-value | Poor surgical outcome | Good surgical outcome | Difference | CI lower | CI upper | *p*-value |
| Average strength | 15.622 | 14.531 | -1.091 | -2.693 | 2.904 | 0.527 | 12.837 | 11.808 | -1.028 | -2.813 | 3.118 | 0.545 |
| Radius | 7.716 | 1.920 | -5.796 | -2.323 | 1.715 | 0.007 | 2.486 | 3.054 | 0.568 | -1.376 | 1.179 | 0.392 |
| Diameter | 9.268 | 3.765 | -5.503 | -3.684 | 3.301 | 0.029 | 3.912 | 4.660 | 0.748 | -2.357 | 2.016 | 0.533 |
| Eccentricity | 8.897 | 2.900 | -5.998 | -3.155 | 2.508 | 0.014 | 3.161 | 3.836 | 0.675 | -1.840 | 1.727 | 0.485 |
| Characteristic path length | 1.916 | 1.508 | -0.409 | -0.493 | 0.420 | 0.208 | 1.740 | 1.938 | 0.198 | -0.603 | 0.529 | 0.526 |
| Global efficiency | 0.790 | 0.731 | -0.059 | -0.125 | 0.120 | 0.489 | 0.656 | 0.600 | -0.056 | -0.126 | 0.129 | 0.483 |
| Local efficiency | 3.019 | 2.419 | -0.600 | -0.827 | 0.821 | 0.240 | 1.957 | 1.663 | -0.294 | -0.737 | 0.692 | 0.554 |
| Clustering coefficient | 0.801 | 0.713 | -0.088 | -0.156 | 0.152 | 0.403 | 0.617 | 0.572 | -0.044 | -0.168 | 0.161 | 0.661 |
| Transitivity | 1.243 | 1.069 | -0.174 | -0.225 | 0.236 | 0.219 | 0.926 | 0.864 | -0.063 | -0.251 | 0.245 | 0.698 |
| Modularity | 0.000 | 0.001 | 0.001 | -0.014 | 0.007 | 0.661 | 0.017 | 0.020 | 0.004 | -0.040 | 0.027 | 0.678 |
| Assortative coefficient | -0.010 | -0.050 | -0.040 | -0.041 | 0.044 | 0.107 | -0.054 | -0.092 | -0.038 | -0.054 | 0.057 | 0.380 |
| Small-worldness index | 0.857 | 0.976 | 0.119 | -0.065 | 0.077 | 0.013 | 0.960 | 0.949 | -0.011 | -0.051 | 0.060 | 0.723 |
|  | **Ipsilateral intrinsic thalamic network** | | | | | | **Contralateral intrinsic thalamic network** | | | | | |
|  | Poor surgical outcome | Good surgical outcome | Difference | CI lower | CI upper | *p*-value | Poor surgical outcome | Good surgical outcome | Difference | CI lower | CI upper | *p*-value |
| Average strength | 15.642 | 13.296 | -2.346 | -3.551 | 3.532 | 0.268 | 10.716 | 12.231 | 1.514 | -3.616 | 3.454 | 0.463 |
| Radius | 3.438 | 2.830 | -0.609 | -6.228 | 4.257 | 0.682 | 3.280 | 3.260 | -0.021 | -1.054 | 1.117 | 0.974 |
| Diameter | 5.548 | 5.141 | -0.407 | -6.916 | 4.363 | 0.641 | 5.211 | 5.927 | 0.716 | -2.006 | 1.767 | 0.488 |
| Eccentricity | 4.792 | 3.757 | -1.035 | -5.611 | 5.253 | *0.001 | 3.993 | 4.402 | 0.409 | -1.521 | 1.228 | 0.560 |
| Characteristic path length | 1.751 | 1.999 | 0.248 | -0.774 | 0.607 | *0.001 | 2.384 | 2.205 | -0.179 | -0.724 | 0.620 | 0.708 |
| Global efficiency | 0.665 | 0.573 | -0.092 | -0.137 | 0.135 | 0.236 | 0.480 | 0.532 | 0.052 | -0.130 | 0.120 | 0.491 |
| Local efficiency | 2.238 | 1.650 | -0.588 | -0.817 | 0.804 | 0.273 | 1.175 | 1.453 | 0.279 | -0.653 | 0.598 | 0.448 |
| Clustering coefficient | 0.645 | 0.529 | -0.115 | -0.161 | 0.176 | 0.243 | 0.425 | 0.497 | 0.072 | -0.146 | 0.148 | 0.448 |
| Transitivity | 0.983 | 0.799 | -0.184 | -0.243 | 0.254 | 0.209 | 0.642 | 0.757 | 0.115 | -0.224 | 0.237 | 0.445 |
| Modularity | 0.014 | 0.042 | 0.028 | -0.040 | 0.032 | 0.147 | 0.106 | 0.039 | -0.067 | -0.076 | 0.059 | 0.121 |
| Assortative coefficient | -0.046 | -0.084 | -0.038 | -0.071 | 0.052 | 0.367 | -0.103 | -0.025 | 0.078 | -0.082 | 0.073 | 0.100 |
| Small-worldness index | 0.976 | 0.952 | -0.024 | -0.116 | 0.118 | *0.001 | 0.949 | 0.966 | 0.017 | -0.043 | 0.055 | 0.685 |

CI: 95% confidence interval of differences between groups

* *p*<0.004
